# Respiratory Illness and Allergy Related to Work and Home Environment among Commercial Pilots

**DOI:** 10.1371/journal.pone.0164954

**Published:** 2016-10-14

**Authors:** Xi Fu, Torsten Lindgren, Gunilla Wieslander, Christer Janson, Dan Norbäck

**Affiliations:** 1 Occupational and Environmental Medicine, Department of Medical Sciences, Uppsala University, University Hospital, Uppsala, Sweden; 2 Respiratory, Allergy and Sleep Research, Department of Medical Sciences, Uppsala University, University Hospital, Uppsala, Sweden; National Taiwan University College of Public Health, TAIWAN

## Abstract

The aim was to study associations between work and home environment and prevalence and incidence of respiratory health and a history of atopy in a 3-y cohort of commercial pilots. A questionnaire was mailed in 1997 to all pilots in a Scandinavian airline company (N = 622); 577 (93%) participated. The same questionnaire was sent to the participants 3 years later, 436 participated (76%). There were questions on asthma, respiratory symptoms and infections, allergies, the cabin environment, psychosocial environment and the home environment. Associations were analyzed by multiple logistic regression, calculating odds ratios (OR) with 95% confidence intervals (95%CI). The incidence of doctors’ diagnosed asthma and atopy were 2.4 and 16.6 per 1000 person years, respectively. Pilots changing type of flight during follow-up got more airway infections (OR = 11.27; 95% CI 2.39–53.14). Those reporting decreased work control (OR = 1.85; 95% CI 1.03–3.31 for 1 unit change) and those with environmental tobacco smoke (ETS) at home (OR = 3.73; 95% CI 1.09–12.83) had a higher incidence of atopy during follow up. Dampness or mould at home was associated with a higher prevalence of asthma symptoms (OR = 3.55; 95% CI 1.43–8.82) and airway infections (OR = 3.12 95% CI 1.27–7.68). Window pane condensation in winter at home, reported at baseline, was associated with increased incidence of asthma symptoms (OR = 4.14; 95% CI 1.32–12.97) and pilots living in newer buildings at baseline had a higher incidence of airway infections (OR = 5.23; 95% CI 1.43–19.10). In conclusion, lack of work control and ETS at home can be a risk factors for development of allergic symptoms in pilots. Window pane condensation at home can be a risk factor for incidence of asthma symptoms. Dampness and mould at home can be a risk factor for prevalence of asthma symptoms and airway infections and living in newer buildings can be a risk factor for incidence of airway infections.

## Introduction

There has been concern about work related diseases among pilots due to their special work condition. Previous epidemiological studies among commercial pilots have studied cancer [1], cardiovascular disease [2], sleeping disorders [3], and medical symptoms on eyes, nose, skin and general symptoms [4–7]. Some studies have investigated respiratory health and allergy among pilots. Two studies on mortality among commercial pilots from UK and Greece reported that standardized mortality ratio due to respiratory illness among pilots was lower than in the general population [8] [9]. A study on morbidity among pilots from New Zealand reported that pilots have a lower prevalence of asthma (4.2%) as compared to the general population (9.6%) in New Zealand [10]. Allergic rhinitis is a common disease among pilots, which can increase the risk for acute sinusitis [11]. Air recirculation has been suggested as a risk factor for airway infections in aircraft, but one study found the same prevalence of airway infections among pilots on flight with cabin air re-circulation (10.6%) and without cabin air re-circulation (10.1%) [6]. One Danish study reported a mean value of 1.6 airway infections (range 0–8) per year among Danish pilots, and almost half of the pilots continues their flight duty without reporting the infection [12]. There are few studies about prevalence or incidence of asthma, allergies, bronchitis, and airway infections among commercial airline pilots.

The aircraft cabin is a crowded indoor environment and there has been concern about spread of airway infections in aircraft. Nowadays the pilots spend all time in the cockpit which has a separate ventilation system. However, when this study was performed, before the 11^th^ September terrorist attack, pilots could spent shorter periods during cruise in the forward part of the cabin socializing with the cabin attendants. Existing studies on spread of infections in aircraft have mainly focused on transmission among passengers and airline crews. Airborne pathogen transmission were reported to be associated with sitting within two rows of the index person on board during more than 8 hours [13]. The transmission is believed to be by large droplets, and increased ventilation rate can decrease the infection risk [13]. Spread of influenza follows previously observed transmission model, but passengers sitting further away than two rows can be infected [13]. Transmission of severe acute respiratory syndrome (SARS) can occur on board faster and at wider areas than the typical transmission model [14]. One tracing contact study of SARS among 1766 passengers found no spread of SARS [15]. Another study of SARS infection traced 304 passengers and crew with one or two index person on board, and reported 23 infections [14]. The relative risk of passengers sitting within three rows in front of the index person was 3.1, compared to passengers seated elsewhere in the cabin [14]. Two tracing contact studies of the swine-origin influenza A (H1N1) pdm09 from UK and US have reported an attack rate of 4.3% [16] and 5.2% [17] respectively among passengers from flights carrying one index passenger, but no crew got infected [17]. The transmission during air travel is a common concern, since it contributes to inter-continental spread of pathogens.

The indoor environment in the cockpit is different from the cabin environment, since the ventilation system in the cockpit is separated from the cabin. Only few studies have investigated the cockpit environment. The cockpit ventilation flow is high (usually 60–80 L/s of outdoor air per person) and there is no air recirculation [6, 18]. The cockpit relative air humidity is typically below 10% during cruise [19], and the temperature is around 22–26°C[19, 20]. Sometimes the cockpit temperature varies because of poor control [20]. One study found that the mean concentration of carbon dioxide in the cockpit is usually around 500–700 ppm, indicating a sufficient ventilation rate [19]. The mean concentrations of nitrogen dioxide and ozone in the cockpit were 7 μg/m3 and 26.3 μg/m3 respectively, and the highest level of ozone was 76.1 μg/m3 [19]. One study reported that the ozone level in an aircraft with an old ozone converter could be similar to that in the aircraft without an ozone converter [21]. The level of formaldehyde in the cockpit was under the detection limit (<5 μg/m3) [19].

It is well known that furry pet allergens can be transported from homes by furry pet keepers to crowded public places, such as schools and day care centres, through clothes and hairs [7], Aircraft are crowded indoor environments but there is little information on the spread of furry pet allergen to the aircraft environment. Furry pet allergens in settled dust in the indoor environment can cause allergic reactions among sensitized subjects. Our previous study investigated levels of furry pet allergens and fungal DNAs by analysing dust collected from the cockpit as well as from the aircraft cabin. Furry pet allergen levels were found in elevated levels in the cockpit as well as in the cabin environment. The mean concentration of cat (Fel d1), dog (Can f1), and horse (Equ cx) allergens in the cockpit were 3.5μg, 2.4μg, and 10.8μg per gram dust respectively [22]. The concentration of total fungal DNA in the cockpit was 3.86*10^4^ cell equivalents (CE) per gram dust. The concentration of *Aspergillus/Penicillium* DNA and *Aspergillus versicolor* DNA were 6.14*10^3^ CE/g and 44 CE/g respectively [22].

The role of the psychosocial work environment have been studied among pilots with respect musculoskeletal symptoms and sleeping problems [23]. Psychosocial stress can increase the risk for airway infections, especially among men [24]. However, we found no studies on associations between the psychosocial work environment and respiratory illness among pilots. Besides the psychosocial work environment, the ban of smoking on board for Scandinavian airlines started from 1 Sep, 1997. Before that smoking was allowed and the level of respirable particles was high in the cockpit environment [19, 25]. Beneficial effects of ban of smoking on board with respect to eye symptoms and tiredness among pilots has been reported [25], but we found no previous studies evaluated the effects of ban of smoking on board with respect to respiratory illness and allergies among pilots.

The first aim of this study was to investigate the prevalence and 3-year incidence of doctors’ diagnosed asthma, asthmac symptoms, bronchitis, nonspecific hyperreactivity, airway infections and pollen or furry pet allergy among commercial airplane pilots. The second aim was to study associations between prevalence and incidence of asthma symptoms, bronchitis, nonspecific hyperreactivity, respiratory infections and pollen or furry pet allergy (a history of atopy) and flight type (long/short-haul flight), psychosocial work conditions (work satisfaction, demand, control, social support), exposure to environmental tobacco smoke (ETS) onboard and selected home environment factors. The home environment factors included type of dwelling, age of the dwelling, ETS at home, window pane condensation in winter and dampness or indoor mould. Health associations were investigated for the prevalence of environmental risk factors at baseline as well as changes of these risk factors during the follow-up period.

## Material and Methods

### Ethics statement

The study procedure and study protocol were approved by the Regional Ethical Review Board in Uppsala, Sweden. All participants gave their informed consent. An information letter sent together with the questionnaire stated that if the subjects answered and returned the questionnaire, it meant they had given their informed consent.

### Study population

This follow-up study is a part of a larger project among airline crew at Scandinavian Airlines System (SAS). A self-administered questionnaire was answered by the pilots at baseline (1997) and at follow-up after 3 years (2000). The first questionnaire was mailed to all pilots on duty in SAS in February to March 1997 (N = 622), 577 pilots participated (93%). This period was chosen because it was after the influenza epidemic period in Sweden, but before the pollen season. Three years later, in February to March 2000, the same questionnaire was sent to all pilots who participated in 1997, 436 participated (76%). The cohort of 436 pilots participating twice was the study population.

### Working conditions

All pilots had a rotating work schedule, changing aircrafts from day to day, but they operated the same type of aircraft for a longer period as they were contracted with the company. During Scandinavian and European flights, the following aircraft were used: Fokker F-28, Mc Donnell Douglas DC-9-21/41/81, and Mc Donnell Douglas MD-80/90 series. All intercontinental flights were operated by Boeing 767 series.

When the baseline questionnaire study was performed in 1997, smoking was allowed on all intercontinental flights and on flights to destinations south of the Alps (3–5 h), and to Greenland (5 h), but not on shorter European flights (1–3 h) or Scandinavian domestic flights (0.6–1.5 h). After 1^st^ September 1997, smoking was banned on all flights, but sporadic occupational environmental tobacco smoke (ETS) exposure could occur in other workplace indoor environments (e.g. in meeting rooms in countries where smoking was allowed).

### Questionnaire study and personal factors

The questionnaire consisted of four sets of questions. The first set were about personal factors, including age, gender, smoking habits and allergies. A current smoker was defined as a subject who reported current smoking (>1 cigarette/ day) in the questionnaire, or who had stopped smoking <6 months ago. The pilots were classified as smokers and nonsmokers according to this definition. The second set was about, specific work environment factors for airline crew, including flight type (long-/short-haul flight) and psychosocial work environment. Short haul flights were defined as flights within Europe with duration of less than 7 hours. Long haul flights were defined as intercontinental flights between Scandinavia and America or Asia with flight duration of 7–12 hours. The third set was about current home environment. The fourth set was about asthma, bronchitis, nonspecific hyperreactivity, and airway infections. The questions on asthma and respiratory symptoms were obtained from European Community Respiratory Health Survey (ECRHS) [26], and two Swedish population studies [24, 27]. The questions on psychosocial work conditions were obtained from a standardized indoor questionnaire (MM 040 NA) developed by the Department of Occupational and Environmental Medicine in Örebro University Hospital [28].The questions on specific work environment factors relevant for airline crews were obtained from another questionnaire[6], which was developed by the Clinic for Occupational and Environmental Medicine, Department of Medical Sciences, Uppsala University. The questions about the home environment were obtained from an additional home environment questionnaire developed for the ECRHS adapted for North European home environment conditions [26].

### Asthma, respiratory symptoms and allergy

There were five “yes/no” questions about asthma symptoms with a recall time of 12 months, including wheezing in chest at any time, attack of breathlessness at rest, attacks of breathlessness after exercise, woken up by attacks of breathlessness, and asthma attacks last 12 month. Subjects reporting at least one of these symptoms were defined as having current asthma symptoms. In addition, there were two more “yes/no” questions on ever had doctor diagnosed asthma, and any current medication for asthma (spray, inhalation powder or tablets). Moreover, there were three “yes/no” questions about other respiratory symptoms, including bronchitis (coughing up phlegm often), nonspecific hyperreactivity in eyes or airways (easily irritated in eyes or respiratory tract by cigarettes smoke, exhaust or solvents), and airway infections (often having common cold and other respiratory infections). Besides, there were two “yes/no” questions on hay fever/pollen allergy and allergy to furry pets respectively. A history of atopy was defined as reporting allergy to pollen or furry pets (cat or dog).

### Work environment and psychosocial work conditions

Questions on work environment factors included long/short haul flight in last three months and psychosocial work conditions. Working on long-haul flights at baseline was an indicator of environmental tobacco smoke (ETS) exposure on board. There were four questions covering different aspects of the psychosocial work conditions, a simplified version of the Swedish demand-control-social support model [29, 30]. The question ‘‘interesting/stimulation at work” measure work satisfaction. The question ‘‘opportunity to influence working conditions” measure the degree of influence on working conditions, and the question ‘‘Do you get help from your colleagues when you have a problem at work” measure the degree of social support. Finally there was a question on ‘‘too much work to do”, which covered stress due to an excess of work. The questions on psychosocial conditions had four possible answers: ‘‘yes, often”, ‘‘yes sometimes”, ‘‘no, seldom”, and ‘‘no, never”.

### Home environmental factors

Information on the current home environment included type of home (single-family house, apartment, other), ownership (own house, own apartment, rented apartment) construction year of the building, year moving to the current home, furry pet keeping, and environmental tobacco smoke (ETS). There were three levels for construction year, including “before 1960”, “1960–1975”, and “after 1975”. Moreover, there were yes/no questions about the indoor painting and redecoration last 12 months, window pane condensation in winter, and four yes/no questions on water damage, visual moulds, signs of floor dampness (bubbles on vinyl floor or blackened parquet) and mould odour at home the last 12 months. The four questions on dampness were combined to one yes/no variable, with coded as “yes” if there was at least one sign of dampness and as “no” if there were no signs of dampness.

### Statistical analysis

For all yes/no questions, no was coded “0” and yes was coded “1”. For the psychosocial questions, “no, never” was coded “3”, “no seldom” was coded “2”, “yes sometimes” was coded “1” and yes, “often” was coded “0”. For the question about ‘‘too much work to do”, the values were assigned the reverse way. The values were then divided by 3, in order to obtain four psychosocial variables each ranging from 0–1, with values either 0, 1/3, 2/3 or 1, where 0 is the most favourable condition and 1 is the most unfavourable condition.

The questions on asthma symptoms consisted of five questions, including wheeze, attacks of breathlessness at rest, attacks of breathlessness after exercise, woken up by attacks of breathlessness, asthma attacks last 12 months. A dichotomous variable of “asthma symptoms” was created, coded as yes if there was a “yes” answer to at least one of these questions and coded as “no” if there were no asthma symptoms. Differences in baseline prevalence of symptoms and exposures between participants and nonparticipants were calculated by Chi-2 test. Differences in age or years of employment, between participants and nonparticipants, were calculated by Student’s t-test. Difference in prevalence of symptoms and exposure at baseline and follow-up were compared by McNemars’s test and Wilcoxon signed rank test.

For work and home exposure, we created new variables measuring the change of exposure, by subtracting the baseline exposure from the exposure at follow up. For a dichotomous exposure variable, the variable was coded “-1” if the exposure occurred at baseline but not at follow up, was coded “0” if the variable did not change between baseline and follow up, and was coded “1” if the exposure occurred only during follow up. The change of psychosocial variables were coded in a similar way, and had a range -1 to 1, after dividing the score by a factor 3.

The incidence of asthma symptoms, doctors’ diagnosed asthma and a history of atopy was calculated, excluding subjects with the particular symptom at baseline. Moreover, since asthma symptoms can occur as a relapse of previous asthma, e.g. childhood asthma, we made additional calculations of incidence of doctors’ diagnosed asthma and asthma symptoms, excluding subject reporting that they ever had asthma.

Since all dependent variables were dichotomous variables, we used multiple logistic regression analysis. Initially, cross-sectional analysis was performed by forward stepwise logistic regression (Wald), including independent variables with p<0.1. One stepwise regression model was created for the prevalence of each of the four dependent variables (asthma symptoms, bronchitis, nonspecific hyperreactivity and airway infections). Baseline work and home environment factors and gender, age, atopy and smoking habit were included initially. In the final mutually adjusted models, confounders (age, gender, smoking and atopy) were always kept in the models irrespectively of their statistical significance, as well as exposure variables with a p-value <0.1. As a next step, associations between incidence of the four health variables were analysed by forward stepwise logistic regression (Wald), including independent variable with p<0.1. Baseline work and home environment factors, changes of work and home environment factors and gender, age, atopy and smoking habit were included initially. In the final mutually adjusted models, confounders (age, gender, smoking and atopy) were always kept in the models irrespectively of statistical significance, as well as exposure variables with a p-value <0.1. Finally, stepwise forward logistic regression analysis (Wald) were performed for prevalence and incidence of self-reported atopy, using the same models and procedures as for the four respiratory health variables. The only difference was that atopy was not included as a confounder.

All statistical calculations were done by SPSS version 21, and a p-value <0.05 was considered statistically significant. Odds ratio (OR) with a 95% confidence interval (CI) was calculated for the logistic regression models. Associations for age in the logistic regression was expressed for an escalation of 10 years.

## Results

**Fig 1:**
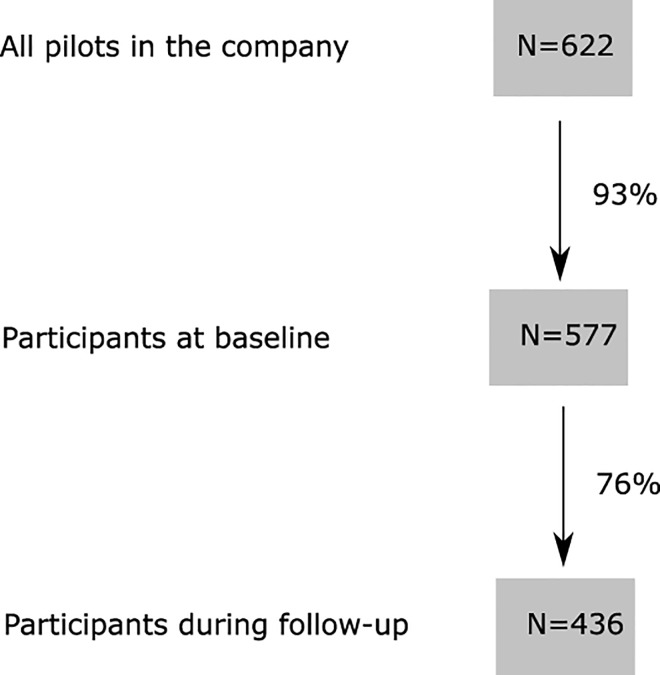
Flow chart of the study population.

Details on the study population is given in Fig 1. Initially, the prevalence of respiratory health symptoms and allergies at baseline were compared between nonparticipants (N = 141) and participants (N = 436) (Table 1). Nonparticipants had a higher prevalence of current wheeze and nonspecific hyperreactivity, but for other health variables there were no significant differences between nonparticipants and participants.

**Table 1:** Prevalence of demographic data, allergies and respiratory illness among nonparticipants at baseline, participants at baseline and participants at follow-up.

Respiratory health variable	Nonparticipants at baseline (N = 141) (%)	Participants at baseline (N = 436) (%)	P-value^a^	Participants at follow-up (N = 436) (%)	P-value^b^
Female gender	4.3	5.3	0.63	5.3	1.00
Current smoker	9.9	12.1	0.45	8.4	0.33
Ever had asthma	0.7	2.6	0.19	2.3	1.00
Doctors’ diagnosed asthma	0.7	1.4	0.53	2.5	0.06
Pollen allergy	12.9	16.9	0.29	19.4	0.06
Furry pet allergy	7.9	10.3	0.41	10.8	0.75
Wheeze or whistling in the chest last 12 months	14.3	7.6	**0.02**	5.7	0.27
Current bronchitis	13.0	8.1	0.23	7.1	0.76
Nonspecific hyperreactivity	30.7	19.8	**0.007**	21.7	0.34
Airway infections	13.5	10.7	0.35	9.7	0.62

a. Comparing prevalence among nonparticipants and participants at baseline, calculated by Chi square analysis for 2*2 tables.

b. Comparing prevalence among participants at baseline and at follow-up, calculated by McMemar test.

Nonparticipants were 3 years older (p = 0.001) and had worked 3 years longer at SAS (p = 0.02).

When comparing work factors, nonparticipants reported less work satisfaction at baseline and less exposure to ETS at work, especially on short haul flights, as compared to participants (Table 2). The prevalence of long haul flights during last 3 months was 25.9%.

**Table 2:** Prevalence of work environment factors among nonparticipants at baseline, participants at baseline and participants at follow-up.

Work factor	Nonparticipants at baseline (N = 141) (%)	Participants at baseline (N = 436) (%)	P-value ^a^	Participants at follow-up (N = 436)	P-value ^b^
**Stimulation**					
Often	73.8	83.1	**0.045**	87.4	0.008
Sometimes	19.9	13.9		11.9	
Seldom	5.0	2.8		0.7	
Never	1.4	0.2		0	
**Demand**					
Never	8.5	5.3	0.37	5.1	0.56
Seldom	46.8	53.8		52.9	
Sometimes	41.8	38.3		38.8	
Often	2.8	2.5		3.2	
**Control**					
Often	13.4	8.6	0.12	11.1	<0.001
Sometimes	24.8	31.1		38.0	
Seldom	43.3	46.6		41.7	
Never	18.4	13.7		9.3	
**Support**					
Often	44.0	50.5	0.39	52.5	0.02
Sometimes	35.8	32.6		35.8	
Seldom	11.4	11.9		8.4	
Never	8.2	5.0		3.3	
**Type of flights** ^**c**^					
Short haul flights without ETS	73.0	63.1	**0.03**	NA	NA
Short haul flight with ETS	9.2	17.9		NA	
Long haul flight with ETS	17.7	19.0		NA	

a. Comparing prevalence among nonparticipants and participants at baseline, calculated by Chi square analysis for 2*4 tables or 2–2 tables (long haul flight).

b. Comparing prevalence among participants at baseline and at follow-up, calculated by Wilcoxon signed rank test.

c. The recall time for the type of flights was last 7 days.

For home environment factors, there were no significant differences between nonparticipants and participant, but a tendency of more furry pet keeping among nonparticipants (p = 0.06) (Table 3). Moreover, the nonparticipants were on average older and had worked longer time with the airline company. Them mean age was 45 y (SD = 10) among nonparticipants and 42 y (SD = 8) among nonparticipants (p = 0.001). The number of years employed at the current airline company was 15 y (SD = 11) among nonparticipants and 12 y (SD = 8) among participants (p = 0.02).

**Table 3:** Prevalence of current home factors among nonparticipant at baseline, participants at baseline and participants at follow-up.

Home factor	Nonparticipants at baseline (N = 141) (%)	Participants at baseline (N = 436) (%)	P-value^a^	Participants at follow-up (N = 436)	P-value^b^
**Construction year**					
Before 1960	29.5	31.3	0.82	34.3	0.52
1961–1975	27.3	23.8		20.9	
After 1975	43.2	44.9		44.8	
**Multifamily house**	19.9	21.9	0.68	15.8	**<0.001**
**Furry pet keeping**	29.1	21.3	**0.06**	26.5	**0.001**
**ETS at home**	9.3	8.3	0.95	4.6	**0.001**
**Indoor painting last 12 months**	23.4	26.3	0.49	26.7	1.00
**Dampness/mould last 12 months**	5.7	7.6	0.44	6.0	0.39
**Window condensation in winter**	11.4	13.7	0.49	11.8	0.21

^a^Comparing prevalence among nonparticipants and participants at baseline, calculated by Chi square analysis for 2*2 tables or 2*3 tables (construction year)

^b^Comparing prevalence among participants at baseline and at follow-up, calculated by McMemar test or Wilcoxon signed rank test.

When comparing changes in prevalence of health factors between baseline and follow up, no significant net-changes were observed (Table 1). However, most of the psychosocial work conditions had improved during the three year follow up period, except for the demand variable which was not changed (Table 2). The pilots tended to move from multifamily houses to single-family houses and had less ETS and less furry pets at home during follow up (Table 3).

Further calculations of number of new cases and the 3-year incidence of the health variables are presented in Table 4. At baseline, totally eleven pilots (2.5%) reported that they had ever had asthma (lifetime incidence) and six of them had the asthma diagnosed by a doctor before the study started. Six of the eleven persons with asthma at baseline (55%) reported that their first asthma attack occurred when they were 1–10 years old (childhood asthma). Two of the eleven pilots with asthma at baseline got his asthma diagnosed by a doctor during the follow up period and three other pilots without asthma at baseline got a new asthma diagnosis by a doctor during follow up (incidence cases).The prevalence of doctors’ diagnosed asthma at baseline and during follow-up were 1.4% and 2.5% respectively and the incidence of doctors diagnosed asthma was 2.4 per 1000 person·years. Totally 5.3% were females. Prevalence of current smoking at baseline was 9.9%, and the prevalence of current smoking at follow-up was 8.4%. There were 10 new smokers and 16 pilots had been giving up smoking. In total, 17 pilots developed atopy during follow-up, and the incidence rate of atopy was 16.6 per 1000 person·years. The prevalence of exposure to personal, work and home environmental factors at baseline and follow-up were published in our previous study [4].

**Table 4:** Number of new cases and symptom incidence during follow up.

Outcome	N^c^	N ^d^	Number of new cases	3-year incidence (%)
**Wheezing in chest any time last 12 months**	**403**	**396**	**16 (12)** ^**e**^	**4.0 (3.0)** ^**e**^
**Attacks of breathlessness at rest last 12 months**	**434**	**423**	**1 (1)** ^**e**^	**0.2 (0.2)** ^**e**^
**Attacks of breathlessness after exercise last 12 months**	**428**	**418**	**5 (4)** ^**e**^	**1.2 (1.0)** ^**e**^
**Woken up by attacks of breathlessness last 12 months**	**431**	**420**	**1 (1)** ^**e**^	**0.2 (0.2)** ^**e**^
**Asthma attacks last 12 months**	**435**	**425**	**0 (0)** ^**e**^	**0 (0)** ^**e**^
**Asthma symptoms**^**a**^	**395**	**388**	**18 (14)** ^**e**^	**4.6 (3.6)** ^**e**^
**Doctors’ diagnosed asthma**	**430**	**425**	**5 (3)** ^**e**^	**1.2 (0.7)** ^**e**^
**Bronchitis**	**395**		**20**	**5.1**
**Nonspecific hyperreactivity**	**345**		**40**	**11.6**
**Airway infections**	**385**		**17**	**4.4**
**A history of atopy**^**b**^	**342**		**17**	**5.0**

a. Asthma symptoms: wheezing in chest at any time, attack of breathlessness at rest, attacks of breathlessness after exercise, woken up by attacks of breathlessness, and asthma attack last 12 month.

b. Pollen or furry pet allergy.

c. Number of participants without particular symptom at baseline.

d. Number of participants without particular symptom at baseline, and the subjects who had ever had asthma at baseline (N = 11) were excluded.

e. Data in parenthesis refers to number of new cases and incidence of asthma symptoms when subjects who had ever had asthma at baseline (N = 11) were excluded.

Table 5 presents the cross-sectional analysis for the baseline data. Women reported more nonspecific hyperreactivity (p = 0.013), while smokers reported more bronchitis (p = 0.027), but less nonspecific hyperreactivity (p = 0.033). Older pilots reported less airway infections (p = 0.004) and less atopy (p = 0.004). Pilots with atopy reported more nonspecific hyperreactivity (p = 0.026). Pilots who reported dampness/mould at home in last 12 months reported more asthma symptoms (p = 0.006) and more airway infections (0.013). Pilots who kept furry pets at home reported less atopy (p = 0.018). Psychosocial work conditions or ETS exposure in the aircraft at baseline were not significantly associated with any of the four investigated health variables.

**Table 5:** Associations between symptom prevalence and baseline exposure (N = 436) ^b^.

Selected variables ^a^	adj OR(CI 95%)	p-value
***Asthma symptoms***		
**Women**	**2.82 (0.96–8.28)**	**0.059**
**Dampness/ mould last 12 months**	**3.55 (1.43–8.82)**	***0*.*006***
		
***Bronchitis***		
**Current smoking**	**2.65 (1.12–6.92)**	***0*.*027***
		
***Nonspecific hyperreactivity***		
**Women**	**3.32 (1.29–8.56)**	***0*.*013***
**Current smoking**	**0.21 (0.05–0.88)**	***0*.*033***
**Atopy**	**1.91 (1.08–3.38)**	***0*.*026***
**High demand**	**1.32 (0.89–1.95)** ^c^	**0.17**
		
***Airway infections***		
**Age**	**0.51 (0.32–0.81)** ^d^	***0*.*004***
**Dampness/ mould last 12 months**	**3.12 (1.27–7.68)**	***0*.*013***
***A history of atopy***		
**Age**	**0.63 (0.45–0.86)** ^d^	***0*.*004***
**Furry pet keeping**	**0.41 (0.19–0.86)**	***0*.*018***

a. The variables were selected by Wald stepwise logistic regression, and the cut-off p-value for the inclusion of variables in the model was 0.1. The stepwise logistic regression model for prevalence of asthma symptoms, bronchitis, nonspecific hyperreactivity and airway infections included following candidate variables: age, gender, atopy, smoking habits; work-related factors: flight type, stimulation at work, work demand, work control, support at work; home environment factors: construction year, multifamily/house, furry pet keeping, ETS at home, indoor painting last 12 months, dampness/mould last 12 months, window condensation in winter. The stepwise logistic regression model for atopy included all the factors included in the other models except atopy.

b. The associations between health variables displayed in this table were calculated by a mutual logistic regression model separately, including the selected independent variables for each health variable, adjusted by age, gender, and smoking habit.

c. For the psychosocial variables, OR was calculated for one step on the scale (0–1).

d. For the variable of age, OR was calculated for each escalation of 10 years.

The longitudinal analysis is presented in Table 6. Older pilots (p = 0.011) had a lower incidence of airway infections. The baseline exposure of window pane condensation (p = 0.015) were positively associated with the incidence of asthma symptoms. The variable of “ever had window pane condensation” (p = 0.030) either at baseline or during follow-up were included in the model for the incidence of asthma symptoms and change of exposures, and it was positively associated with the incidence of asthma symptoms. Pilots who changed type of flight during follow-up (p = 0.002), either from long-haul to short-haul or from short-haul to long-haul, had a higher incidence of airway infections than those who continued with the same type of flight. Pilots reporting low psychosocial support at work at baseline had lower incidence of atopy, though the significance level is on the border (p = 0.06). Pilots who reported lower control at work (p = 0.039) during follow-up had more onset of atopy. Pilots living in newer dwellings at baseline (p = 0.012) had a higher incidence of airway infections. ETS at home at baseline (p = 0.010) was positively associated with new onset of atopy. The variable of “ETS at home ever” either at baseline or during follow-up (p = 0.005) was positively associated with new onset of atopy. There was no association observed for incidence of bronchitis and nonspecific hyperreactivity. There were no association between ETS exposure in the aircraft at baseline and incidence of any of the four health variables.

**Table 6:** Associations between symptom 3-year incidence and baseline exposure and change of exposure ^b^.

Selected variables ^a^	adj OR(CI 95%)	p-value
***Asthma symptoms***		
**Window pane condensation at baseline**	**4.14 (1.32–12.97)**	***0*.*015***
***Airway infections***		
**Age**	**0.33 (0.14–0.77)** ^**d**^	***0*.*011***
**Construction year of dwelling**	**5.23 (1.43–19.10)**	***0*.*012***
**Flight type change**	**11.27 (2.39–53.14)**	***0*.*002***
**Construction year change**	**0.19 (0.04–0.81)**	**0.025**
***A history of atopy***		
**Low support**	**0.39 (0.15–1.03)** ^**c**^	**0.06**
**ETS at home at baseline**	**3.73 (1.09–12.83)**	***0*.*010***
**Low control change**	**1.85 (1.03–3.31)** ^**c**^	***0*.*039***

a. The variables were selected by Wald stepwise logistic regression, and the cut-off p-value for the inclusion of variables in the model was 0.1. The stepwise logistic regression model for incidence of asthma symptoms, bronchitis, nonspecific hyper-reactivity and airway infections included variables stating baseline exposure and the change during follow-up of following factors: age, gender, atopy, smoking habits; work-related factors: flight type, stimulation at work, work demand, work control, support at work; home environment factors: construction year, multifamily/house, furry pet keeping, ETS at home, indoor painting last 12 months, dampness/mould last 12 months, window condensation in winter. The stepwise logistic regression model for atopy included all the factors included in the other models except atopy.

b. The associations between symptoms displayed in this table were calculated by a mutual logistic regression model separately, including the selected independent variables for each health variable, adjusted by age, gender, and smoking habit.

c. For the psychosocial variables, OR was calculated for one step on the scale (0–1).

d. For the variable of age, OR was calculated for each escalation of 10 years.

## Discussion

Our study followed pilots in a Scandinavian airline company over three years. The home environment seemed to be the most important indoor environment with respect to prevalence and incidence of respiratory illnesses and self-reported allergy. Dampness/mould at home was a risk factor for prevalence of asthma symptoms and window pane condensation in winter was a risk factor for incidence of asthma symptoms. Moreover, living in a newly constructed home was associated with a higher incidence of airway infections and pilots exposed to environmental tobacco smoke (ETS) at home had a higher incidence of atopy. We could find a few associations between working conditions and health. Changing type of flight (long or short haul flight) was associated with new onset of airway infections and those reporting that they got less control of their working conditions during the follow up developed more atopy (self-reported pollen or furry pet allergy).

The study has some strengths and limitations. It is based on historical data for pilots from one airline company collected around 15 years ago. The study is unique and we found no previous longitudinal study on development of asthma, bronchitis, nonspecific hyperreactivity and airway infections among pilots. However, the number of incident cases was relatively low, which limits the power of the study. Despite this limitation we found a number of significant associations but sometimes with wide confidence intervals. Moreover, the study only covered pilots from one airline company which could limit the generalizability of the study. Another limitation is that we did not have any clinical tests to diagnose airway infections, asthma or pollen or furry pet allergy (self-reported atopy). However, some validation studies on questionnaire data on asthma and allergy are available. A review article concluded that self-reported data on doctors’ diagnosed asthma has high specificity (94%) when validating against clinical diagnosis but lower sensitivity (68%) [31]. Three European validation studies of self-reported allergy to pollen or furry pets in adults, using objective allergy testing as gold standard, found good specificity (89–97%) but lower sensitivity (28–55%) for self-reported allergy [32–34] Thus a majority of those reporting doctors’ diagnosed asthma, pollen or furry pet allergy could be expected to have asthma or atopic sensitization (allergy), and an underestimation rather than an overestimation of the true prevalence could be expected.

Epidemiological studies can be influenced by selection bias and information bias (recall bias). The study population consisted of all Stockholm based pilots in one Scandinavian airline company. The participation rate was 93% at baseline investigation, and 76% of those participants at baseline. Despite the relatively high participation rate, we found that nonparticipants were older and had a higher prevalence of wheeze and nonspecific hyperreactivity at baseline, as compared to participants. Moreover, nonparticipants were less satisfied with their working conditions and were less exposed to ETS on board at baseline. These results indicate a health based selection where more healthy pilots tended to join the cohort, which could lead to an underestimation of the risks of the work exposure. However, for home environment exposure, we found no difference between participants and nonparticipants. The questionnaire included questions on the symptoms, as well as questions about the work environment and the home environment. This may introduce recall bias in the cross-sectional analysis. However, in the longitudinal analysis of new onset and development of symptoms over three years, when baseline data on exposure was used, recall bias should not be a major concern.

Changes of work or home environment factors from baseline to follow up is not further discussed since it has been reported in a previous publication from the study [8]. Most of the pilots (94.7%) were men, mean age at baseline was 45 y. At baseline, totally eleven pilots reported that they had ever had asthma and six of them had the asthma diagnosed by a doctor (1.4%). The low prevalence of doctor’s diagnosed asthma could be due to selection since pilots have regular medical examinations, and are not allowed to have severe or uncontrolled asthma. However, mild or moderate asthma is allowed if it is well controlled [35]. The incidence of doctors’ diagnosed asthma was 2.4 cases per 1000 person·years in our study, if we exclude the pilot reporting asthma, not diagnosed by a doctor, at baseline. A Swedish population based cohort study (mean age 34 years at baseline) from 1980–1993 reported that the overall incidence rate of adult onset asthma was 1.1 per 1000 person·years, 1.0 per 1000 person·years among males, and 1.3 per 1000 person·years among females [36]. A study among general Nordic population (mean age 40 years at baseline) during 1999–2001 reported that the incidence of doctor diagnosed asthma was 2.2 cases per 1000 person·years, 1.5 cases per 1000 person·years among males, and 2.9 cases per 1000 person·years among females [37]. Our study was conducted during a similar study period (1997–2000), and the mean age of the pilot cohort was 45 years. Thus we can conclude that the incidence of doctor diagnosed asthma in our study among commercial pilots was similar as in the general population in Northern Europe. In our study, the prevalence of self-reported atopy (pollen or furry pet allergy) was 20.4% at baseline and the incidence rate was 16.6 per 1000 person·years during follow-up. The prevalence among the pilots was higher than the 14% of pollen or furry pet allergy reported from a study from 1997 in a random sample of the Swedish population follow-up [38]. We found no study on incidence of atopy in the general adult population in Sweden from the same time period as our study.

Female pilots had a higher prevalence of asthma symptoms (borderline significance p = 0.059) and significantly more nonspecific hyperreactivity than men. The small proportion of female pilots makes the confidence intervals wide when comparing men and women. The higher prevalence of asthma symptoms among female pilots is in agreement with other population studies reporting a higher prevalence of asthma symptoms in women [37]. One study reported that females have stronger airway response to cigarette smoke, fuels or other pollutants [39] and that they can be more exposed to perfumes, cooking fumes and cleaning detergents [39].

We found that older pilots had less airway infections. We can only speculate on the reason for this association. It could be a selection effect, where pilots who easily get airway infections leave the occupation. It could also be that age is associated with the family situation. Older pilots may have less smaller children at home who can bring infections from schools or day care centres to the family. Moreover, we found that the prevalence of airway infections was positively associated with atopy. We found no previous population study from Sweden on associations between atopy and airway infections among adults. Finally, we found that smokers reported more bronchitis but less nonspecific hyper-reactivity in the cross-sectional study. It is well known that smokers develop bronchitis[40], but the negative association between smoking and nonspecific hyper-reactivity was unexpected. However, the question on nonspecific hyperreactivity asked about irritation easily in eyes or airways when exposed to traffic exhausts, perfume or tobacco smoke and the negative association could be because smokers are more used to environmental tobacco smoke. However, since smokers have a lower ability to smell and taste [41, 42], they may have less ability to perceive sensory irritation from odours or irritants.

Window pane condensation in wintertime was the most consistent environmental risk factor in the home environment. This indicator of dampness was associated with incidence of asthma symptoms. Moreover, change of window pane condensation status was associated with incidence of asthma symptoms. Window pane condensation in winter as a proxy variable for poor indoor environment have been studied in Sweden. It is a sign of damp indoor environment and is a risk factor for growth of house dust mites in homes [43]. Homes with reported window pane condensation in winter has significantly lower air exchange rate, higher relative air humidity and higher levels of house dust mite allergens and total volatile organic compounds (TVOC) as compared to homes without window condensation [44]. Window pane condensation has been reported to be associated with increased prevalence of wheeze in children in Sweden [45] and female university students in Japan [46]. In addition, we asked about presence of signs of dampness and mould the last 12 months in the home [26]. Reports on dampness or mould at home were associated with increased prevalence of asthma symptoms and airway infections. This finding is in agreement with previous studies and a review from WHO have concluded that there is sufficient evidence that dampness and mould is associated with asthma symptoms [47] symptoms. Dampness and mold have also been reported to be associated with respiratory infections [48].

We identified some other significant risk factors in the home environment. We found that those living in newer home buildings (after 1975) had a higher incidence of airway infections. In a previous population study among adults in Sweden, there was an increased prevalence of respiratory infections among adults living in houses constructed from 1976–1985 while no increased prevalence was observed for homed constructed after 1985. In this study, adults living in buildings constructed before 1960 were used as reference category [49]. The drastic increase of the energy price in 1975 initiated measures to reduce the energy consumption in Swedish buildings. Moreover, a self- level mortal containing casein was used in Swedish buildings from 1997–1983. This product caused emission of odorous compounds such as ammonia and 2-acetophenone from the floor [50]. Thus it is possible that the increased prevalence of respiratory infections among pilots living in homes constructed after 1975 was due to energy saving measures as well as the introduction of new building materials, resulting in an impairment of the indoor environment. Moreover, one recent study from South Korea found that moving to a new house may increase the prevalence of asthma symptoms [51].

We have no information in this study on types of building materials in the home, except for the question on recent indoor painting. The issue on health effects of emissions from building materials is a complex issue. In Sweden, some houses are made of wood but most are concrete buildings. Formaldehyde emission form chipboard is not a major problem anymore because of well controlled production of the chipboard, and the mean formaldehyde levels in Swedish homes are low (mean 22 μg/m^3^) [52]. The literature suggests that building dampness, indoor mould and ETS are the most important risk factors for respiratory illness in the home environment and the role of specific chemical emissions from building materials is more unclear [53].

We found that pilots exposed to environmental tobacco smoke (ETS) at home, either at baseline or at follow-up, had an increased incidence of atopy (pollen or furry pet allergy). We have not found any other study on associations between ETS exposure and development of atopy among adults, but a number of studies have demonstrated that ETS exposure in early childhood is associated with increased risk of atopic sensitization [54]. Finally we found that pilots with furry pets at home had a lower prevalence of atopy. This is in agreement with a recent population study among adults in Stockholm, showing a negative association between keeping furry pets and pollen allergy [55]. The most probable reason for the negative association is selection, where allergic persons avoid keeping furry pets.

We found a few associations between occupational risk factors and respiratory health and a history of atopy among the pilots. Change of flight type was associated with increase of new onset of airway infection. Pilots who changed type of flights, both those from long- to short-haul and those from short- to long-haul, reported more airway infections at follow-up. We have no clear explanation to this finding, but it is possible that some pilots change flight type because of some health problems and that these health problems are associated with an increased risk for respiratory infections. We found that pilots who reported less control over the work situation during the follow up developed more atopy. Less control over the work conditions is expected to be a psychosocial risk factor at work. In a previous cross-sectional study on associations between the psychosocial work environment in the Swedish workforce, atopy and asthma symptoms, no associations were found between low control and atopy [24].

In conclusion, the home environment can be an important risk factor with respect to prevalence and incidence of respiratory illness and a history of atopy among commercial pilots. However, some health associations were found for the work environment. Lack of work control can be a risk factor for the development of allergic symptoms in pilots and changing of flight can be a risk factor for developing airway infections. Environmental tobacco smoke at home, window pane condensation in winter, and dampness and mould at home can be risk factors for asthma symptoms, airway infections and allergy among commercial pilots. The home environment should not be neglected when assessing associations between occupational risk factors and respiratory illness.
